# Neuroimaging features in inflammatory myelopathies: A review

**DOI:** 10.3389/fneur.2022.993645

**Published:** 2022-10-18

**Authors:** Laura Cacciaguerra, Elia Sechi, Maria A. Rocca, Massimo Filippi, Sean J. Pittock, Eoin P. Flanagan

**Affiliations:** ^1^Department of Neurology, Mayo Clinic, Rochester, MN, United States; ^2^Neuroimaging Research Unit, Division of Neuroscience, IRCCS San Raffaele Scientific Institute, Milan, Italy; ^3^Vita-Salute San Raffaele University, Milan, Italy; ^4^Neurology Unit, Department of Medical, Surgical and Experimental Sciences, University of Sassari, Sassari, Italy; ^5^Neurology Unit, IRCCS San Raffaele Scientific Institute, Milan, Italy; ^6^Neurorehabilitation Unit, IRCCS San Raffaele Scientific Institute, Milan, Italy; ^7^Neurophysiology Service, IRCCS San Raffaele Scientific Institute, Milan, Italy; ^8^Laboratory Medicine and Pathology, Mayo Clinic, Rochester, MN, United States

**Keywords:** myelopathies, MRI, neuromyelitis optica spectrum disorders, multiple sclerosis, myelin oligodendrocyte glycoprotein, paraneoplastic, transverse myelitis

## Abstract

Spinal cord involvement can be observed in the course of immune-mediated disorders. Although multiple sclerosis (MS) represents the leading cause of inflammatory myelopathy, an increasing number of alternative etiologies must be now considered in the diagnostic work-up of patients presenting with myelitis. These include antibody-mediated disorders and cytotoxic T cell-mediated diseases targeting central nervous system (CNS) antigens, and systemic autoimmune conditions with secondary CNS involvement. Even though clinical features are helpful to orient the diagnostic suspicion (e.g., timing and severity of myelopathy symptoms), the differential diagnosis of inflammatory myelopathies is often challenging due to overlapping features. Moreover, noninflammatory etiologies can sometimes mimic an inflammatory process. In this setting, magnetic resonance imaging (MRI) is becoming a fundamental tool for the characterization of spinal cord damage, revealing a pictorial scenario which is wider than the clinical manifestations. The characterization of spinal cord lesions in terms of longitudinal extension, location on axial plane, involvement of the white matter and/or gray matter, and specific patterns of contrast enhancement, often allows a proper differentiation of these diseases. For instance, besides classical features, such as the presence of longitudinally extensive spinal cord lesions in patients with aquaporin-4-IgG positive neuromyelitis optica spectrum disorder (AQP4+NMOSD), novel radiological signs (e.g., H sign, trident sign) have been recently proposed and successfully applied for the differential diagnosis of inflammatory myelopathies. In this review article, we will discuss the radiological features of spinal cord involvement in autoimmune disorders such as MS, AQP4+NMOSD, myelin oligodendrocyte glycoprotein antibody-associated disease (MOGAD), and other recently characterized immune-mediated diseases. The identification of imaging pitfalls and mimics that can lead to misdiagnosis will also be examined. Since spinal cord damage is a major cause of irreversible clinical disability, the recognition of these radiological aspects will help clinicians achieve a correct and prompt diagnosis, treat early with disease-specific treatment and improve patient outcomes.

## Introduction

Inflammatory myelopathies can be recognized in the context of numerous immune-mediated disorders, including multiple sclerosis (MS), aquaporin-4(AQP4)-IgG-positive neuromyelitis optica spectrum disorders (AQP4+NMOSD), and myelin oligodendrocyte glycoprotein (MOG) antibody-associated disease (MOGAD). The term “myelitis” refers to inflammation of the spinal cord, which can involve its cross-sectional area entirely or partially, hence being defined transverse or partial (1).

Despite the broad spectrum of etiologies, the clinical manifestations of spinal cord dysfunction are stereotyped and include symmetric or asymmetric weakness in the limbs (upper and/or lower, depending on the level of involvement), sensory abnormalities (hypoesthesias, paresthesias, allodynia), bladder or bowel dysfunction (urgency or retention) or sexual dysfunction. Lower-motor neuron signs (fasciculations, hypo/areflexia and flaccid tone) can be observed in association to the above mentioned symptomatology in case of gray matter involvement (2).

Based on the underlying pathophysiology, inflammatory myelopathies can be classified into (i) cell-mediated in the context of autoimmune disorders confined to the CNS (e.g., MS), (ii) associated with specific antibodies primarily targeting CNS antigens, and (iii) associated with systemic autoimmune disorders having secondary CNS involvement (e.g., systemic lupus erythematosus, Sjogren's syndrome, and sarcoidosis) (3). In addition, a novel class of immune-mediated myelopathies, occurring after the administration of drugs affecting the immune system function (e.g., immune checkpoint inhibitors for cancer therapy, and TNF-alfa inhibitors), is emerging (4, 5).

When no specific cause is identified, the term “idiopathic transverse myelitis” or “idiopathic myelitis” is applied. Although the proportion of myelitis of unclear etiology remains high in the population (including patients with clinically isolated myelitis who will later meet MS diagnostic criteria), (6) a former study demonstrated that 70% of patients referred with suspected idiopathic transverse myelitis eventually received a specific myelopathy diagnosis (7). Similarly, the proportion of idiopathic myelopathies was approximately 22% in a Latin American cohort of longitudinally extensive myelopathies (8). Therefore, the term idiopathic must be limited to those cases with a still undetermined etiology after an extensive diagnostic assessment and appropriate follow-up duration.

In this review, we will examine inflammatory myelopathies from an imaging perspective, starting with the classical definition of short and longitudinally extensive sagittal T2-hyperintense lesions, moving to the MRI signs and enhancement patterns associated with specific disorders, and concluding with the chronic evolution of these spinal cord lesions over time. Lastly, we will provide an overview of the main mimics of inflammatory myelopathies and a summary of findings with other imaging techniques.

Before focusing on imaging features of spinal cord lesions, below is a short background on clinical, paraclinical and laboratory investigations, which can assist the diagnosis of inflammatory myelopathies.

## Clinical features assisting the diagnosis of inflammatory myelopathies

In the case of immune-mediated disorders, the temporal evolution of symptoms from onset to nadir, defined “time-to-nadir”, can provide clues on the underlying pathophysiological process, and an acute-subacute presentation (e.g., from one to 21 days) usually suggests an immune-mediated cause (1, 9). This general rule does not always apply to clinical practice, since a hyperacute presentation (<12 h) can be observed in necrotizing paraneoplastic myelopathies, (10) while chronic progressive presentation (e.g., over 21 days) occurs in the case of progressive MS and progressive solitary sclerosis (11). More rarely, a progressive course can be found in association with sarcoidosis, (12) paraneoplastic myelopathies (13) and in the presence of glial-fibrillary acidic protein (GFAP) antibodies (but in this case the myelitis is almost always accompanied by cerebral involvement) (14).

However, taking in mind these possible exceptions, extreme presentations (i.e., acute/hyperacute or progressive) should suggest an alternative non-inflammatory diagnosis. Aside from traumatic causes, abrupt onset usually indicates a vascular etiology, such as in the case of spinal cord infarct. Cases of acute presentations in patients with spinal cord metastases have been reported as well (15).

In contrast, in the presence of a progressive disease course, spondylotic myelopathy, dural arteriovenous fistula, and spinal cord neoplasms should be considered (1). To note, infectious disorders, especially viral (e.g., Varicella-Zoster virus, Herpes simplex, Cytomegalovirus, West Nile virus, human immunodeficiency virus, and enteroviruses), and, more rarely, bacterial or parasitic, can also manifest with subacute symptoms (1).

## Paraclinical non-imaging investigations assisting the diagnosis of inflammatory myelopathies

Beside imaging, paraclinical testing helping in the diagnostic framing of these disorders include cerebrospinal fluid (CSF) examination, and blood tests. For instance, the presence of oligoclonal bands in the CSF and typical brain lesions involving periventricular or juxtacortical areas suggests MS as the most likely disease cause, while absence of oligoclonal bands, very high cellularity in CSF (especially neutrophils or eosinophils) combined with non-specific or normal brain MRI findings could indicate AQP4+NMOSD. Although a systematic description of the pathophysiology, clinical manifestations, and paraclinical tests of the autoimmune disorders associated with inflammatory myelopathies goes beyond the purpose of this review, Table 1 provides a general and non-comprehensive summary of these immune-mediated conditions, as a short background for the understanding of the findings discussed below.

**Table 1:** Clinical and paraclinical features of the main causes of inflammatory myelopathies.

	**Onset**	**CSF**	**Brain MRI**	**Other**
**Multiple sclerosis**	Acute-Subacute/progressive	Frequent oligoclonal bands (>85%); (16) pleocytosis frequent but almost always <50/μL	Periventricular, cortical or juxtacortical and infratentorial lesions; central vein in more than 35–50% of brain white matter lesions (17, 18)	None
**AQP4+NMOSD**	Acute-Subacute/hyperacute	Rare oligoclonal bands (<20%); pleocytosis relatively frequent	Normal or non-specific; typical lesions can be found surrounding the 3rd and 4th ventricle (area postrema) corticospinal tract, and linear ependymal enhancement	Autoimmune comorbidities; strong female predominance
**MOGAD**	Acute-Subacute	Rare oligoclonal bands (<20%); pleocytosis frequent	Ill-defined borders, deep gray matter, white matter, middle cerebellar peduncles, large pons, corticospinal tract, cerebral cortical T2-hyperintensity, leptomeningeal enhancement	Recent vaccination or infection; children particularly predisposed
**Anti-GFAP astrocytopathy**	Acute-Subacute/progressive	Oligoclonal bands may be present (around 50%); pleocytosis frequent	Typical linear perivascular radial enhancement in the white matter perpendicular to lateral ventricles	Coexisting anti-NMDA-R and AQP4 antibodies; possible concomitant ovarian teratoma
**Paraneoplastic myelopathies**	Subacute/progressive	Oligoclonal bands may be present; pleocytosis frequent	Normal	Constitutional symptoms, weight loss, older age
**Sarcoidosis**	Subacute/progressive	Rare oligoclonal bands; pleocytosis extremely frequent	Basilar leptomeningeal enhancement, hydrocephalus, white matter enhancing lesions, involvement of pituitary gland, hypothalamus, and cavernous sinus	Systemic involvement (lungs, eyes, skin, heart)
**Behçet's disease**	Subacute/progressive	No oligoclonal bands; pleocytosis extremely frequent	Large lesions mainly located in the brainstem, internal capsule, and deep gray matter; possible pachymeningeal enhancement	Systemic involvement (oral, genital, skin, eyes)

Only typical findings are reported.

AQP4+NMOSD, aquaporin-4 antibody positive neuromyelitis optica spectrum disorders; CSF, cerebrospinal fluid; GFAP, glial-fibrillary acidic protein; MOGAD, myelin oligodendrocyte glycoprotein antibody-associated disease; MRI, magnetic resonance imaging; NMDA-R, N-methyl-D-aspartate receptor.

Antibody-testing is a powerful tool for the diagnostic assessment of suspected inflammatory myelopathies. Autoantibodies targeting AQP4 and MOG are the most frequent causes of antibody-associated transverse myelitis. In fact, AQP4-IgGs are found in half of the patients with longitudinally extensive spinal cord lesions, and transverse myelitis occurs in 12–56% of patients with MOGAD (19, 20), depending on age and disease duration. Both AQP4-IgG and MOG-IgG should be tested in serum, where sensitivity exceeds CSF, although rare cases of isolated antibody positivity in the CSF have been reported (21, 22). In patients with MOGAD, the presence of MOG-IgG in the CSF usually occurs in patients with spinal cord or brain involvement (23). Higher CSF titers seem to associate with more severe disability, suggesting a prognostic value of CSF antibody testing in this disease (23).

To maximize accuracy, a cell-based assay (CBA) is preferred to enzyme-linked immunosorbent assay (ELISA, to avoid false positives) (24, 25), and should be performed prior to immunotherapy (to avoid false negatives). To note, even with these precautions, low-titer false positive MOG-IgG can be encountered with other neurologic diseases, and clinicians should limit antibody testing to the cases with clinical and MRI features that are suggestive for MOGAD (26, 27). On the contrary, with AQP4-IgG, false positive results are extremely rare when cell-based assays are utilized.

Other antibodies that may be considered, especially but not solely when a paraneoplastic etiology is suspected, include GFAP, amphiphysin, collapsin response mediator protein-5 (CRMP-5), antineuronal nuclear antibodies type 1 and 2 (ANNA-1[anti-Hu] and ANNA-2[anti-Ri]), Purkinje cell cytoplasmic autoantibody type-1 (PCA1/anti-Yo), glycine receptor, and glutamic acid decarboxylase-65 (GAD-65). In these cases serum and CSF testing should be considered complementary (28).

Finally, systemic antibodies such as anti-nuclear (ANA), anti-double strand DNA, anti-extractable nuclear antigens (including Sjogren's syndrome A and B), anti-neutrophil cytoplasmic antibody (ANCA, perinuclear or cytoplasmatic), and anti-phospholipids (e.g., lupus anticoagulant, anti-beta2-glycoprotein-1, anti-cardiolipin) should be tested to assess for systemic autoimmunity.

## Spinal cord MRI protocol and lesion features

On magnetic resonance imaging (MRI), an optimal assessment of the spinal cord requires the acquisition of sagittal and axial T2-weighted images (at least two sequences), and post-contrast T1-weighted images (29). Recommended T2-sequences for a standardized spinal cord visualization are conventional or fast spin-echo and dual-echo fast spin-echo (e.g., proton density and T2-weighted scans) (30). However, dual-echo fast spin-echo is rarely acquired in clinical practice, and a valuable alternative to proton density images is nowadays represented by the short-tau inversion recovery (STIR), that has greater sensitivity to T2-hyperintensities than conventional T2-weighted sequences due to better contrast-to-noise ratio (30, 31). Nonetheless, STIR sequence is also more prone to false-positive findings related to flow-related artifacts, and therefore should be examined in association with another T2-weighted sequence (e.g., conventional/fast spin-echo).

The identification of the spinal cord segment to study can be guided by the clinical examination. However, a complete visualization of the spinal cord is suggested in the diagnostic phase, as the presence of multiple lesions and the regional distribution of lesions provide important diagnostic pieces of information (29). Indeed, although most lesions fall in the cervical and thoracic segments, involvement of the conus (which may be considered as the lumbar segment of the spinal cord) is recognized to associate with prominent erectile or sphincteric dysfunction in MOGAD.

Axial images provide crucial information about lesion characteristics, location (e.g., involvement of the central gray matter or white matter, etc.), and are very useful diagnostically as they can identify lesions not visible on sagittal images. Thus, both sagittal and axial spinal cord images should always be undertaken in patients presenting with myelopathy.

Spinal cord lesions usually appear as single or multiple intramedullary T2-hyperintensities, extending along a variable number of consecutive vertebral segments and involving the gray or white matter alone or in combination. T1-hypointensity can coexist, particularly at 3T (32), and acute lesions are usually characterized by contrast enhancement, sometimes displaying specific patterns.

### Acute phase MRI

Acute spinal cord lesions are usually characterized by cord swelling, edema, and gadolinium enhancement. However, the sensitivity of imaging studies can be hindered in case of early acquisition. For example, the baseline MRI scan in an AQP4+NMOSD patient with intractable vomiting, hemiplegia and thermo-dolorific anesthesia, initially disclosed a tiny hyperintensity in the area postrema, while subsequent scans demonstrated a cervical longitudinally extensive myelitis (33).

#### Sagittal view of inflammatory spinal cord lesions

The main information provided by the visualization of the spinal cord on the sagittal plane are lesion vertebral segment location and length measured by contiguous vertebral body segments.

##### Lesion location: Cervical vs. thoracic

Inflammatory spinal cord lesions involve more often the cervical cord but can also be observed in the thoracic segment and conus. Since any spinal cord segment can be involved, lesion location is not particularly useful in distinguishing between competing myelopathy etiologies. However, conus involvement is a potential clue to MOGAD and should prompt MOG-IgG testing, although it can be encountered also in other myelitis etiologies such as MS (34) as well as in noninflammatory mimics such as spinal dural arteriovenous fistula (35).

##### Lesion length: Short vs. long

A sagittal T2-lesion length cut-off of at least three consecutive vertebral segments defines the presence of longitudinally extensive lesions, as opposed to short lesions. This definition initially proved very useful in discriminating between inflammatory spinal cord lesions due to MS from AQP4+NMOSD. However, there are a wide variety of other neuroinflammatory myelopathic etiologies that can associate with longitudinally extensive T2-lesions, including MOGAD, anti-GFAP encephalomyelitis, paraneoplastic myelopathies, and sarcoidosis.

###### Short lesions

***Multiple sclerosis***. MS represents the leading cause of inflammatory myelopathy. Spinal cord lesions are observed in up to 90% of MS patients (30), usually in the form of multiple short T2-lesions (1). Acute spinal cord lesions in MS can be accompanied by focal edema, and variable patterns of enhancement (more frequently associated with a non-specific nodular or patchy enhancement although ring enhancement when present can be suggestive) (36, 37). The enhancement usually resolves within 8 weeks, whereas the T2-lesion usually persists (1).

***Multiple sclerosis “spectrum”: progressive solitary sclerosis and pure relapsing short*** ***myelitis***. Short spinal cord lesions also occur in progressive solitary sclerosis, a disorder considered along the MS spectrum in which patients usually have a single CNS lesion, generally do not fulfill MS diagnostic criteria for dissemination in space and develop progressive motor disability from that lesion (11).

Short spinal cord lesions can also occur in purely relapsing patients falling under the definition of “pure relapsing short myelitis”. These patients are characterized by at least two episodes of myelitis accompanied by short spinal cord lesions, no evidence of typical MS lesions in the brain (hence not fulfilling the MS diagnostic criteria), and negative antibody-testing (38, 39). Lesions demonstrate a slight predilection for the cervical cord (55%), are usually peripheral posterior, and enhancement is common (45%) (39). Patients usually experience partial rather than complete myelitis, have a positive response to MS disease-modifying drugs, and undergo a secondary progression in around 17% of cases (39). Cells and proteins in the CSF are normal or mildly elevated, and oligoclonal bands are present (38, 39). Since all these features closely resemble MS, some Authors refer to this phenotype as “pure spinal MS” (38). However, other characteristics significantly deviate from classical MS, including a female-to-male ratio of five, and the absence of significant brain involvement despite over 3 years of follow-up (39), therefore future studies are warranted to clarify the pathophysiological definition of this entity.

***Atypical causes of short spinal cord lesions: aquaporin-4-IgG-positive Neuromyelitis*** ***optica spectrum disorders and myelin oligodendrocyte glycoprotein-antibody-associated*** ***disease***. The presence of short lesions does not necessarily exclude alternative diagnoses, since short T2-hyperintense lesions, often centrally located and accompanied by concomitant T1-hypointensity, have been observed in 14%-15% of AQP4+NMOSD patients at disease onset (40, 41) and in 10% of Latin American NMOSD patients with spinal cord involvement (42).

In this case, red flags arguing against MS include non-Caucasian ethnicity, the presence of tonic spasms (e.g., short paroxysmal episodes of involuntary painful contractions of limbs or truncal muscles, sometimes triggered by movement or hyperventilation), evidence of coexisting autoimmunity (e.g., myasthenia gravis, connective tissue disorders, thyroiditis), the lack of CSF oligoclonal bands, brain MRI not suggestive of MS, and lesions that extend 2–2.5 vertebral segments (40). However, 92% of AQP4+NMOSD patients with initially short myelitis had a subsequent longitudinally-extensive lesion (40).

Short lesions have also been described in around 20% of patients with MOGAD, where the central location, the uncommon presence of gadolinium enhancement during the acute phase, together with other clinical and brain MRI features, can help in the differential diagnosis (34, 43).

###### Long lesions

The presence of extensive spinal cord involvement opens the door to a large number of diagnoses, such as AQP4+NMOSD, MOGAD, sarcoidosis, connective tissues disorders, paraneoplastic myelopathies, and other myelopathies associated with CNS-specific autoantibodies. Therefore, in this situation, the acquisition of axial images and post-contrast sequences is particularly relevant. The features of longitudinally extensive lesions on the axial plane are discussed in detail in the section “Axial view of inflammatory spinal cord lesions”.

***Aquaporin-4-IgG positive neuromyelitis optica spectrum disorders***. The most common cause of myelitis accompanied by a longitudinally extensive T2-lesion is AQP4+NMOSD, especially in case of recurrent longitudinally extensive transverse myelitis (up to 90% of cases) (44). Acute long lesions in AQP4+NMOSD can show focal cord swelling, concomitant T1-hypointensity (70%) (45) and gadolinium enhancement (up to 90%) (46). Longitudinally extensive leptomeningeal enhancement can be observed as well and usually corresponds to the level of the parenchymal enhancement (47). The frequency of T1-hypointense signal is more common than in MS (around 20%) and other causes of longitudinally extensive spinal cord lesions (45), and may be associated with higher degree of pain (48). Sometimes, cervical lesions can extend cranially up to the area postrema, as described in 19% of AQP4+NMOSD patients with cervical longitudinally-extensive lesions. However, this finding is not specific for AQP4+NMOSD and can be encountered with other myelopathy etiologies (49). AQP4+NMOSD lesions usually enhance, and contrast enhancement can be longitudinally extensive, patchy, or ring-like; the presence of the latter can help discriminating from other etiologies (37, 50). Enhancement persistence beyond 3 months is uncommon (2).

The presence of longitudinally extensive spinal cord lesions favors the diagnosis of NMOSD over MS, (34, 51, 52) but not MOGAD (34). In contrast, multiple longitudinally extensive lesions favor MOGAD over AQP4+NMOSD (34).

***Myelin oligodendrocyte glycoprotein antibody-associated disease***. In patients with MOGAD, myelitis are observed in around 12% of children and 26% of adults (19), and longitudinally extensive lesions represent approximately 60–80% of spinal cord lesions (34, 43). Myelitis lesions can occur in isolation or together with brain or optic nerve involvement (e.g., in the course of an ADEM manifestation of MOGAD). More rarely, concomitant spinal cord and peripheral nerve involvement occurs in MOGAD as myeloradiculitis, which is detected at thoraco-lumbar MRI by the presence of diffuse enhancement (53) and thickening (54) of the spinal nerve roots.

Even during the acute phase, parenchymal cord enhancement in MOGAD is less common than in MS or AQP4+NMOSD. When present, it is often faint, (34, 55) while spinal cord leptomeningeal enhancement may be more common (55). Since lesions are usually centrally-located, they can appear as linear on sagittal view, defining the “ventral sagittal line” appearance (34). MOGAD myelitis can be accompanied by “pseudo-dilatation” of the central canal within the acute lesion, which mimics physiological central canal dilatation (56).

***GFAP-antibody-associated myelitis***. GFAP-antibody-associated astrocytopathy is a newly described autoimmune disorder with onset more frequent in adults over 40 years of age a slight predilection for females (14, 57). Although the most common clinical manifestations are encephalitis and meningoencephalitis, spinal cord involvement is common, as observed in approximately 22–68% of patients (14, 57). Myelopathy is usually subacute and can occur alone (11%) or in association with encephalic or meningeal inflammation (i.e., encephalomyelitis, 8% and meningoencephalomyelitis, 3%) (14). MRI usually reveals a hazy ill-defined longitudinally extensive T2-lesions in the spinal cord with punctate (50%), central canal (30%) and pial or leptomeningeal (40%) enhancement (35). Concomitant brain abnormalities are almost invariably present (83%), and cauda equina enhancement with thickening can occur as well (20%) (35). The presence of other accompanying symptoms, including encephalopathy, tremor and visual disturbances due to optic disc edema should lead to clinical suspicion. CSF findings usually reveal increase white blood cells and proteins, with oligoclonal bands occurring in about half of patients (14, 35). In 22% of these patients there is an underlying neoplasm identified, most often an ovarian teratoma (14).

***Paraneoplastic myelopathies***. Paraneoplastic myelopathies are usually accompanied by longitudinally extensive T2-lesions with a linear appearance on sagittal images, although normal spinal cord MRI may also occur (13). Paraneoplastic myelopathies usually have a subacute or insidious onset and progressive course, mimicking primary progressive MS. Rare reports of acute necrotizing paraneoplastic myelopathies also exist. In patients with myelopathy and coexisting cancer, a neural autoantibody is detected in around 80% of cases (13), with amphiphysin (24%) and CRMP-5 (16%) being most common (13), followed by ANNA-1/anti-Hu and ANNA-2/anti-Ri, PCA1/anti-Yo, and GAD-65 (58). In 60% of cases the neurologic manifestations precede cancer detection, and primary treatment relies on the eradication of the underlying neoplasm. The most frequently associated cancers are lung carcinoma (primarily small cell type), breast adenocarcinomas, and thymomas. Given the strong association with cancer, surveillance imaging is warranted every 6 to 12 months when the initial cancer screening is negative (13). The motor outcome is usually poor and most patients become non-ambulatory and wheelchair-dependent (13). To note, cases of paraneoplastic AQP4+NMOSD have been reported, (59–61) especially in the elderly or in middle-aged males (62, 63). Therefore, screening for neoplasms should be performed in the AQP4+NMOSD patients at disease onset meeting these demographic red flags.

***Inflammatory myelopathies during immune checkpoint inhibitors***. This is a newly recognized class of immune-mediated myelopathies, occurring after the administration of drugs affecting the immune system function. Immune checkpoint inhibitors are aimed at promoting antitumor surveillance by inhibiting the molecules involved in the negative regulatory steps of immune system activation, including cytotoxic T lymphocyte associated antigen 4 (CTLA-4), and the programmed death-1 receptor (PD-1) and its ligand PD L1. These drugs may trigger CNS autoimmunity by unmasking a pre-existing subclinical autoimmune diathesis or by favoring antitumor response against antigens expressed in both the tumor and the CNS, hence leading to a paraneoplastic syndrome (64, 65). These myelopathies can occur in isolation or associated with other neurological manifestations, usually after three to seven cycles of treatment (66). Although disability at nadir is generally severe, the administration of high-dose steroids and other immunotherapies leads to improvement in about half of patients (66). Radiological findings are heterogeneous and include MS-like focal lesions, longitudinally extensive lesions, diffuse white matter tract hyperintensities with enhancement, and atrophy. Autoantibodies in serum or CSF have been reported in some cases including CRMP-5, ANNA-1, GFAP, and AQP4 (66–68).

***Sarcoidosis***. Spinal cord involvement in the course of sarcoidosis warrants emphasis because a myelopathy can be the presenting feature of the disease and can mimic AQP4+NMOSD in clinical practice (12, 46, 69). Sarcoid spinal cord lesions are usually (but not exclusively) longitudinally-extensive (45–77%) (12, 70) with concomitant spinal cord swelling, but clinical manifestations are milder than those observed in AQP4+NMOSD or MOGAD-associated myelitis (clinical-MRI dissociation) (1). Other imaging patterns are the spinal meningitis or meningoradiculitis and the anterior myelitis associated with areas of disc degeneration and ventral subpial enhancement, which account for 23 and 10% of cases, respectively (12). During the acute phase enhancement is observed in almost all patients (12). On sagittal images, gadolinium enhancement is often linear dorsal subpial, extending two or more vertebral segments with or without pial involvement and possible extension in the central canal. Leptomeningeal enhancement can also be observed at the level of the cauda equina and spinal nerve roots (71).

***Behçet's disease***. Lesions of variable length can be rarely observed during Beçhet's disease, although are more commonly long and with a specific appearance on axial images defined “bagel sign” (discussed below). Leptomeningeal enhancement is observed in around 30% of patients (72). Beçhet's disease is more common in people originating from the Mediterranean regions (especially Turkey), the Middle-East and the Far East, grossly corresponding to the Old Silk Road (73), probably due to common environmental and genetic background including the frequency of the HLA B51. Neurological involvement usually occurs in the fourth decade of life and is almost three times higher in men compared to women. Concomitant systemic manifestations are frequently observed and include the presence of oral or genital ulcers, dermatologic issues (e.g., papulopustular lesions, erythema nodosum, and pathergy reaction), and uveitis. CSF examination usually reveals a moderate increase of proteins, neutrophilic pleocytosis (up to 400 cells/μl) and no oligoclonal bands (74). Brain MRI usually reveals large lesions preferentially located in the brainstem, deep gray matter and, to a lesser extent, white matter; pachymeningeal enhancement can be observed (75).

***Rheumatologic disorders***. Other systemic autoimmune disorders with potential for secondary CNS involvement such as systemic lupus erythematosus and Sjogren's syndrome, may present with myelopathy and spinal cord lesions. AQP4+NMOSD and, to a lesser extent, MOGAD, can coexist with these autoimmune disorders. In addition, AQP4-IgG have been documented in around 75% of patients with connective tissue disorders and longitudinally extensive transverse myelitis, (76) although this frequency might be lower in Latin American patients (77). Therefore, while myelitis can be a manifestation of a connective tissue disorder, it is mandatory to assess for AQP4-IgG in all cases, as most will be due to coexisting AQP4+NMOSD rather than to the connective disease itself.

In addition, antiphospholipid syndrome coexisting with AQP4+NMOSD may increase the risk of thrombotic complications including deep venous thrombi particularly given the concomitant risk factors including immobility with myelitis, potential dehydration from area postrema syndrome and central-line associated thrombi in those receiving plasma exchange (78).

Patients with inflammatory myelopathies associated with systemic lupus erythematosus usually present with acute flaccid symmetric paraparesis, hypoesthesia, and sphincteric dysfunction preceded by headache; concomitant optic neuritis is common, and the nadir of symptoms is usually reached within 1 to 2 days (77).

***Post-infective or post vaccinial myelopathies***. Post-infective or post-vaccinal myelopathies can also associate with longitudinally extensive lesions and are more common in children (79). Spinal cord involvement occurs in around 25% of patients with ADEM (80), but up to half of patients with ADEM have MOG-IgG and thus ADEM should be considered a syndrome rather than its own disease. Finally, during the ongoing COVID-19 pandemic very rare cases of myelitis following SARS-CoV-2 infection or vaccination have been reported, although given the frequency of SARS-CoV-2 infection and vaccination in the population it is difficult to be definitive about any association. Some reports have highlighted a radiological pattern characterized by patchy longitudinally extensive lateral or dorsal column tractopathies sometimes with corresponding enhancement (81, 82).

***Atypical causes of long spinal cord lesions: Multiple sclerosis***. The presence of longitudinally extensive spinal cord lesions is rare in MS, with a frequency of 0–5% in adults (8, 51, 83) and 5–14% in pediatric patients (55, 84). However, a spinal cord evaluation entirely relying on the sagittal view might lead to an overestimation of longitudinally extensive lesions in MS, since multiple short lesions can coalescence and appear as one, while the axial evaluation reveals separated hyperintensities (83). Nonetheless, a diffuse T2-hyperintensity along the entire spinal cord can be noted in patients with primary progressive MS (85) or with secondary progressive MS (86), particularly when using proton-density images (87).

#### Axial view of inflammatory spinal cord lesions

The evaluation of spinal cord lesions on the axial plane allows the localization of the pathological process in the white matter and/or gray matter. Also, radiological signs associated with specific inflammatory myelopathies have been described when examining the spinal cord in a transverse section.

##### Lesion location: White vs. gray matter

Wedge-shaped white matter involvement, especially in the lateral or dorsal columns is typically seen in MS lesions, although anterior columns or central gray matter involvement can be observed rarely. Paraneoplastic myelopathies usually show a selective and specific involvement of the white matter tracts and are usually located at the lateral or dorsal columns. Gadolinium enhancement can also be tract-specific, helping distinguish from nutritional deficiency myelopathies (vitamin B12, folate, copper) which similarly involve the tracts but rarely enhance. This pattern is detected in 30 to 50% of paraneoplastic myelopathies (13).

Central pure gray matter involvement or mixed gray-white matter involvement, with lesions affecting more than half of the cross-sectional area of the cord is found in AQP4+NMOSD, MOGAD, and sarcoidosis.

##### MRI clues to diagnosis visible on the axial plane

Several novel radiological signs have proved useful for the differential diagnosis of inflammatory myelopathies, as described below.

###### H-sign

A selective involvement of the gray matter, hence outlining a H-shape, is observed in about 30% of patients with MOGAD and in a minority of patients with AQP4+NMOSD (<10%), but not in patients with MS (34). This pattern has also been reported with acute flaccid myelitis after enterovirus infections and West Nile virus encephalomyelitis (88, 89). Spinal cord infarct can show similar MRI findings (90, 91). One possible caveat is that lesions in MOGAD can also be diffuse and not necessarily restricted to the gray matter, similar to AQP4+NMOSD (34).

###### Brighter spotty lesions

Spinal cord lesions with intrinsic areas characterized by a T2-signal intensity at least equal or greater to the intensity of CSF have been described in around 30–50% (92–94) of AQP4+NMOSD myelitis but are rarely if ever found in MS and idiopathic transverse myelitis (93, 94). Studies in MOGAD are conflicting, with some reporting its presence (92) and others not (95, 96). Concomitant T1-hypointensity can be observed, but it must be milder than that of the CSF to differentiate from syringomyelia (93). Although initially believed to be due to tissue damage and necrosis, all these lesions were found to disappear after the acute phase, suggesting they might reflect reversible local changes associated with acute inflammation, such as blood-spinal-cord barrier breakdown (93). Despite the greater potential for assisting the diagnosis of inflammatory myelopathies, their presence in around 30% of spinal cord infarcts (97) highlights the importance of considering that disorder when the myelopathy is hyperacute.

###### Bagel sign

On axial imaging, spinal cord lesions of variable sagittal length associated with Bechet's disease can have a T2-hyperintense appearance with a hypointense center and possible gadolinium enhancement at the periphery. This bagel-like aspect has been observed in 93% of Beçhet-associated myelopathies and might reflect the presence of venous engorgement and/or blood products accumulation within the spinal cord (72).

#### Enhancement patterns of inflammatory spinal cord lesions

Specific patterns of gadolinium enhancement have proven helpful in the correct identification of the diagnosis associated with longitudinally extensive lesions, as demonstrated by a recent study where, in a blinded setting, a trained neurologist and a trained neuroradiologist were required to identify the diagnosis of 113 cases of longitudinally extensive lesions based on features of T2-weighted and post-gadolinium T1-weighted images. The correct etiology was identified more often using post-gadolinium T1-weighted images (84–88%) when compared to T2 weighted images (54–60%), further highlighting the utility of post-gadolinium images in the evaluation of myelopathy (98).

The majority of acute lesions due to MS or AQP4+NMOSD are accompanied by gadolinium enhancement, whereas this is less frequent in MOGAD (34).

##### Ring enhancement

Complete or incomplete ring enhancement occurs in a similar proportion of spinal cord lesions in patients with MS and AQP4+NMOSD (up to 30%), therefore its diagnostic utility for the discrimination of these disorders is limited, especially on axial view (37). On the other hand, it is rarely encountered in MOGAD, (1) and apparently not observed in sarcoidosis-associated myelitis (37). Given the significant cranio-caudal extension of spinal cord lesions in AQP4+NMOSD, ring enhancement can appear lens-shaped or ellipsoid on sagittal view (elongated wing enhancement), which is not seen in MS lesions.

##### Tract specific enhancement

Is associated with paraneoplastic myelopathies and may overlap with the spinal cord T2-hyperintensity which is also typically tract-specific (13).

##### Linear dorsal subpial enhancement and trident sign

The presence of linear dorsal subpial enhancement extending for at least two consecutive vertebral segments on sagittal view has been described in patients with sarcoidosis (46); concomitant central canal enhancement can occur. On the axial plane, it usually extends inward, outlining a trident shape, in the so-called “trident sign” (99). This pattern of enhancement can be reliably identified amongst a group of myelopathies (98). A ventral subpial enhancement with a “braid-like” aspect has also been described in patients with sarcoidosis (100).

**Figure 1:**
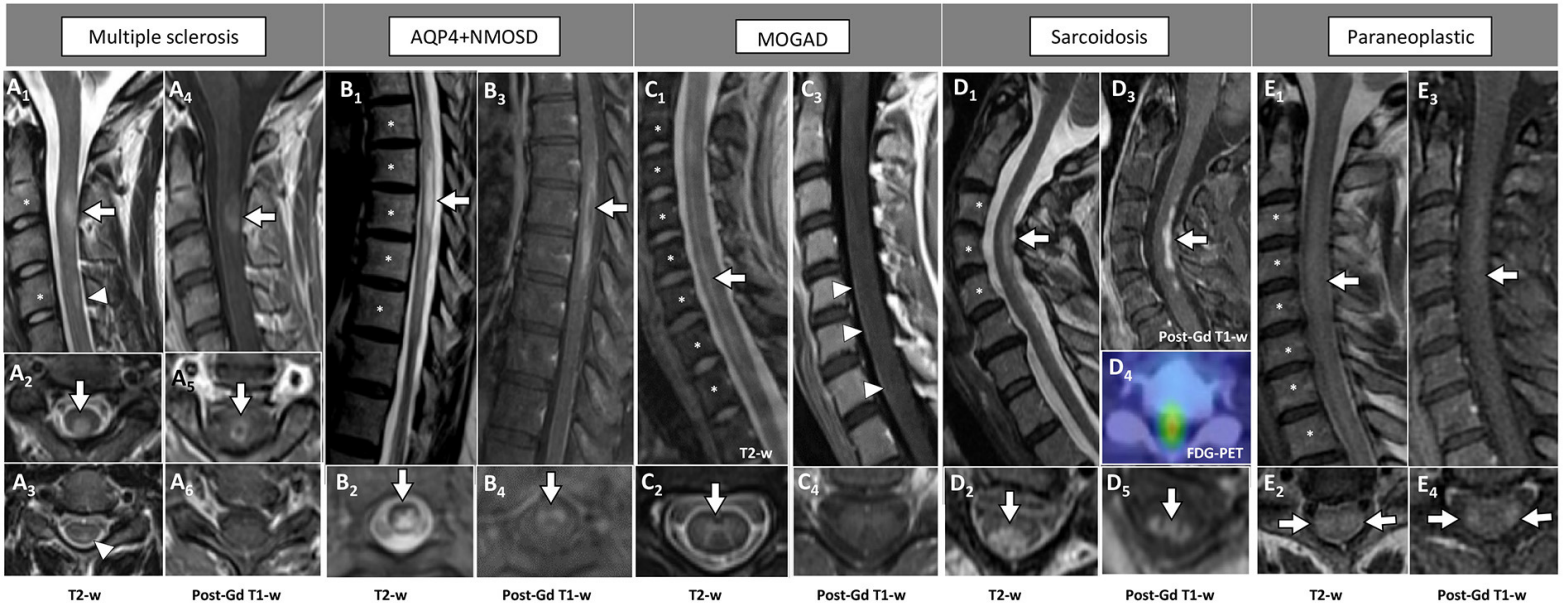
Examples of inflammatory myelopathies on MRI. The extension of the T2-hyperintensities along consecutive vertebral segments is highlighted by asterisks. [**(A)** MS] Acute short cervical lesion with focal spinal cord swelling [**(A_1_)** arrow] involving the central gray matter and the dorsal columns [**(A_2_)** arrow], showing homogeneous enhancement on sagittal view [**(A_4_)** arrow] and a circular ring of enhancement on axial view [**(A_5_)** arrow]. A chronic lesion at the level of C4 is also observed [**(A_1_)** arrowhead], located in the dorsal columns [**(A_3_)** arrowhead] and not showing contrast enhancement **(A_6_)**. [**(B)** AQP4+NMOSD] Thoracic spinal cord lesion, longitudinally extensive on T2-weighted images [**(B_1_)** arrow], centrally located [**(B_2_)** arrow], showing lens-shaped ring enhancement on sagittal view [**(B_3_)** arrow], and inhomogeneous enhancement on axial view [**(B_4_)** arrow]. [**(C)** MOGAD] Cervico-thoracic spinal cord lesion, longitudinally extensive on T2-weighted images characterized by a “sagittal linear” aspect [**(C_1_)** arrow] corresponding to a selective involvement of the gray matter (“H-sign”), on axial view [**(C_2_)** arrow]. Parenchymal enhancement is absent **(C**_**3**_**,C**_**4**_**)**, although a subtle leptomeningeal enhancement is detected [**(C**_**3**_**)** arrowheads]. [**(D)** Sarcoidosis] Cervical spinal cord lesion, longitudinally extensive on T2-weighted images [**(D**_**1**_**)** arrow] involving the central gray matter and the dorsal columns [**(D**_**2**_**)** arrow], with dorsal enhancement on sagittal view [**(D**_**3**_**)** arrow] and “trident sign” on axial view [**(D**_**5**_**)** arrow]. Increased glucose uptake is also observed, corresponding to an area of red color in the positron-emission tomography **(D**_**4**_**)**. [**(E)** Paraneoplastic] Cervical spinal cord lesion, longitudinally extensive on T2-weighted images [**(E**_**1**_**)** arrow] involving the lateral white matter tracts bilaterally [**(E**_**2**_**)** arrows], showing subtle enhancement on sagittal view [**(E**_**3**_**)** arrow] and axial view [**(E**_**4**_**)** arrows]. FDG-PET, ^18^F-Fluorodeoxyglucose-positron emission tomography; Gd, gadolinium; T2-w, T2-weighted; T1-w, T1-weighted.

Examples of acute inflammatory spinal cord lesions are shown in Figure 1.

Table 2 summarizes the main radiological features of inflammatory myelopathies.

**Table 2:** Summary of radiological features in the main causes of inflammatory myelopathies.

	**Acute phase**								**Chronic phase**		
	**Spinal cord lesion–Sagittal view**			**Spinal cord lesion–Axial view**			**Enhancement**		**Spinal cord lesion**	**Spinal cord volume**	**Enhancement**
	**Length of T2-lesion^a^**	**Location**	**Clues to diagnosis**	**Location**	**Central canal involvement**	**Clues to diagnosis**	**Parenchymal**	**Leptomeningeal**	**Evolution**	**Atrophy**	**Time to resolution**
**Multiple sclerosis**	Short	Cervical or thoracic	Multiple short T2-lesions	Peripheral (dorsal/lateral columns often involved)	No	Multiple peripheral cord lesions	Homogeneous or ring	No	Lesions can reduce in size but rarely resolve	Yes	Less than three months
**AQP4+** **NMOSD**	Long	Cervical or thoracic	Cord swelling	Central (both gray and white matter)	Yes	Bright(er) spotty lesions	Patchy or ring (lens-shaped)	Rare	Possible fragmentation of long lesions, can occasionally resolve	Yes	Less than three months
MOGAD*	Long; Long and short	Cervical or thoracic, conus frequently involved	Linear appearance (“ventral sagittal line”)	Central (can be gray matter restricted with a H-sign)	Yes	H-sign	May or may not be present; faint	Yes	Lesions often completely resolve	No	Less than three months
**Anti-GFAP astrocytopathy**	Long	Cervical or thoracic, conus possible	Ill-defined	Central	Yes	Concomitant encephalitis	Multifocal punctate	Yes	Lesions usually improve	Can be present	Unknown
Paraneoplastic myelopathies*	Long	Cervical or thoracic	Linear appearance	Peripheral (dorsal/lateral columns)	No	Tract-specific	Tract-specific	No	Unknown	Unknown	Unknown
**Sarcoidosis**	Long, but short can also occur	Cervical or thoracic	Cord swelling	Central (both gray and white matter)	Yes	None	Dorsal-subpial on sagittal and when combined with central canal can form axial “trident sign”	Yes	Lesions usually improve	Can be present	Reduces with treatment but can take six months or longer to resolve completely
**Behçet's disease**	Long, but short is not uncommon	Cervical or thoracic	None	Peripheral with hypointense center	No	Bagel sign	Peripheral, especially located in the thoracic cord	Yes	Lesions can completely resolve	Can be present	Unknown

Only typical findings are reported.

The symbol * indicates disorders with possible negative MRI.

AQP4+NMOSD, aquaporin-4-IgG-positive neuromyelitis optica spectrum disorders; GFAP, glial-fibrillary acidic protein; MOGAD, myelin oligodendrocyte glycoprotein antibody-associated disease.

^a^Long, ≥3 vertebral segments; Short, <3 vertebral segments.

### Chronic phase or follow-up MRI

Despite an adequate imaging and an extensive diagnostic assessment, the etiology of spinal cord lesions after the acute phase may remain undefined. Follow-up MRI examinations might provide additional information for a correct diagnosis and give insight into the pathogenesis. Lesions may remain stable or undergo progressive reduction, fragmentation, or resolution with late development of focal or diffuse cord atrophy. With MS, MOGAD and AQP4+NMOSD, gadolinium enhancement usually resolves within 3 months.

In patients with MS the extent of spinal cord T2-hyperintensity usually reduces in size but rarely resolves completely, (101) while contrast enhancement invariably resolves completely.

#### Reduction, fragmentation, and resolution

Three months after the acute episode, longitudinally extensive lesions in AQP4+NMOSD can undergo subsequent fragmentation into multiple short lesions (estimated around 35% of AQP4+NMOSD cases with myelopathy) (102) and possible resolution (5%) (103). A former study suggested that a complete resolution of spinal cord lesions during the disease course may indicate a subset of patients characterized by favorable prognosis, since this feature was encountered in around 70% of patients with a benign phenotype (103).

However, when a complete resolution of the lesion is observed, the diagnosis of MOGAD should be suspected first. In fact, complete lesion resolution was described to occur in around 80% of MOGAD cases, with complete normalization of spinal cord findings (101).

After immunotherapy improvement of spinal cord lesions was also reported in patients with anti-GFAP astrocytopathy (35) and Behçet's disease (72). Interestingly, although details on frequencies are not reported, MRI abnormalities seem to completely reverse in some patients with Behçet's disease (72).

#### Persistence of enhancement

Persistent enhancement at two-month follow-up since clinical presentation was described in more than 90% of patients with sarcoidosis, in contrast with 12% of AQP4+NMOSD (46). Indeed, during sarcoidosis, spinal cord enhancement may persist for over 6 months and take a year or longer to disappear completely (2).

#### Focal atrophy

Unequivocal focal spinal cord atrophy at sites of previous inflammatory demyelination can be observed in MS (104) and AQP4+NMOSD (105). Atrophy has also been reported in patients with anti-GFAP associated astrocytopathy (57), sarcoidosis (106), and Behçet's disease (72), but data on larger cohorts are required to draw solid conclusions on its frequency. Focal atrophy may be particularly evident in those cases of progressive solitary sclerosis or patients who have unilateral progression from a single CNS demyelinating lesions along the lateral columns (11, 107).

### Myelitis with normal MRI

A case series reported that during the acute phase spinal cord MRI can be negative in around 10% of patients with MOGAD-associated myelitis despite a severe clinical presentation (e.g., required assistance to ambulate). Spinal cord lesions became overt in half of patients after a median follow-up of 6 days (108), suggesting that, in cases of clinical-radiological mismatch, repeat spinal cord imaging should be encouraged. Negative MRI was also reported in one patient with anti-GFAP antibody associated myelitis (14).

It is notable that absence of T2-hyperintensities is reported also in up to 50% of paraneoplastic myelopathies (13) and in some cases of spinal cord involvement during Sjogren's disease, systemic lupus erythematosus, and vasculitis (109). Antibodies directed against glycine receptor (110, 111) and glutamic acid decarboxylase-65 (GAD65) (112) can be associated with myelopathies in the setting of stiff-person syndrome or its variants with cerebral involvement (e.g., progressive encephalomyelitis with rigidity and myoclonus [PERM]). Anti-amphiphysin antibodies can be associated with a paraneoplastic stiff-person syndrome (113). With stiff-person syndrome and its variants, the spinal cord MRI can be normal (111, 112) and electromyography can assist with diagnosis. Among noninflammatory causes, an initial normal MRI can also occur with spinal cord infarct (91). Genetic disorders (e.g., hereditary spastic paraplegias, adrenomyeloneuropathy, Friedreich's ataxia), and chronic infections (e.g., human T-cell lymphotropic viruses) are usually accompanied by progressive spinal cord atrophy, in the absence of focal visible lesions (109).

## Mimics and pitfalls

The differential diagnosis of inflammatory myelopathies includes vascular, compressive, neoplastic, infectious, metabolic, nutritional, and genetic causes.

**Figure 2:**
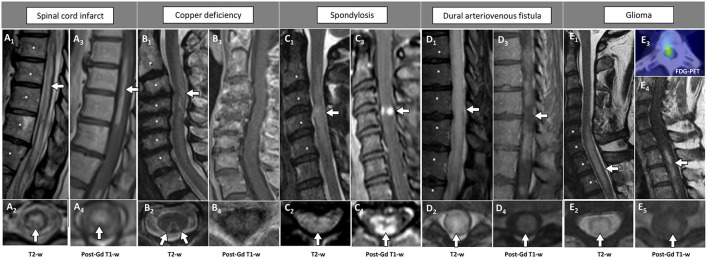
Examples of longitudinally extensive spinal cord lesions mimicking inflammatory myelopathies on MRI. The extension of the T2-hyperintensities along consecutive vertebral segments is highlighted by asterisks. [**(A)** Spinal cord infarct] Thoracic T2-hyperintense spinal cord lesion [**(A_1_)** arrow] mainly involving the gray matter [**(A_2_)** arrow], with corresponding anterior linear enhancement on sagittal view **(A_3_)** and homogeneous enhancement on axial view **(A_4_)**. [**(B)** Copper deficiency] Cervical T2-hyperintense spinal cord lesion [**(B_1_)** arrow] selectively involving the dorsal columns bilaterally in a rabbit ears configuration [**(B_2_)** arrows], not showing concomitant contrast-enhancement **(B**_**3**_**,B**_**4**_**)**. [**(C)** Spondylosis] Cervical T2-hyperintense spinal cord lesion located right under the level of maximal spinal cord compression [**(C_1_)** arrow], diffusively involving the cross-sectional area of the cord [**(C_2_)** arrow], showing a transverse area of contrast-enhancement on sagittal view [“pancake sign”, **(C**_**3**_**)** arrow], corresponding to a peripheral area of contrast enhancement on axial images [**(C**_**4**_**)** arrow]. [**(D)** Dural arteriovenous fistula] Thoracic T2-hyperintense spinal cord lesion [**(D**_**1**_**)** arrow], diffusively involving the cross-sectional area of the cord [**(D**_**2**_**)** arrow], showing a focal lack of enhancement in an otherwise homogeneous pattern of contrast enhancement on sagittal images [“missing-piece sign”, **(D**_**3**_**)** arrow], and homogeneous enhancement on axial images [**(D**_**4**_**)** arrow]. [**(E)** Glioma] Cervico-thoracic T2-hyperintense spinal cord lesion [**(E**_**1**_**)** arrow], diffusively involving the cross-sectional area of the cord [**(E**_**2**_**)** arrow], showing homogeneous contrast enhancement on both sagittal [**(E**_**4**_**)** arrow], and axial images [**(E**_**5**_**)** arrow]. Increased glucose uptake is also observed, corresponding to an area of red signal in the positron-emission tomography **(E**_**3**_**)**. FDG-PET, ^18^F-Fluorodeoxyglucose-positron emission tomography; Gd, gadolinium; T2-w, T2-weighted; T1-w, T1-weighted.

The main radiological features associated with non-inflammatory mimics of inflammatory myelopathies are summarized in Table 3, while MRI examples of these disorders are shown in Figure 2.

**Table 3:** Radiological features of the main mimics of inflammatory myelopathies.

	**Spinal cord lesion–Sagittal view**			**Spinal cord lesion–Axial view**		**Enhancement**	**Differential diagnosis**		
	**Length**	**Location**	**Aspect**	**Location**	**Aspect**		**Inflammatory mimic**	**Diagnostic pitfalls**	**Other clinical clues to diagnosis**
**Spinal cord infarction**	Variable	Cervical or thoracic	Linear “pencil-like”; restricted diffusion; vertebral body infarct in 10%;	Central or anterior	T2-hyperintensity of the anterior horn cells (owl or snake-eye sign) or H sign; concomitant vertebral artery occlusion	Owl or snake-eye sign; linear strip of anterior enhancement	AQP4+NMOSD	Bright(er) spotty lesions; Owl or snake-eye sign	Hyperacute presentation with flaccid paraparesis preceded by back pain
							MOGAD	H-sign; linear aspect	
**Nutritional deficits (B12, copper)**	Long	Cervical or thoracic	Linear dorsal cord	Peripheral	Tract-specific	Usually absent	Paraneoplastic myelopathies	Long tractopathy	Progressive course; lack of gadolinium enhancement
**Spondylotic myelopathy**	Variable	Cervical	Fusiform spindle-shaped	Central	Nonspecific	Transverse band of flat pancake enhancement; involving the periphery and sparing the gray matter; slow resolving enhancement after treatment	Nonspecific (inflammatory myelopathy)	Presence of enhancement	Progressive course
**Spinal DAVF**	Long	Thoracic, conus	Homogeneous hyperintensity centrally located; serpentine/ dilated veins/flow voids	Central	T2-hypointense flow voids along conus/cord surface; parenchymal T2-hyperintense signal sparing periphery	Patchy, homogeneous with an area of lack of enhancement “missing piece sign”, or absent enhancement; enhancement of large veins	Progressive MS	Progressive course	Progressive course; worsened with Valsalva and steroids
							MOGAD, AQP4+NMOSD	Conus involvement; long lesion	
**Spinal ependymoma**	Long	Cervical or thoracic, conus	Cord swelling, regular margins; rostral or caudal cyst; T2-hypointense rim, cap sign	Central	Nonspecific	Homogeneous non-specific	AQP4+NMOSD, sarcoidosis	Long lesion with cord swelling	Progressive course
**Spinal astrocytoma**	Long	Cervical or thoracic, conus	Cord swelling, irregular margins	Peripheral	Excentric with irregular margins and edema	Heterogeneous or no enhancement	MOGAD	Pediatric age, conus involvement	Progressive course
**Spinal metastases**	Variable	Cervical or thoracic	T2-hyperintensity largely exceeding the enhancing area	Central or peripheral	Nonspecific	Rim and flame sign: ring enhancement surrounding a region with fainter contrast-enhancement, flame-shaped appearance of enhancement rostral or caudal to the rim; dot sign: punctate enhancement in the center of the lesion on axial view	AQP4+NMOSD	Enhancement pattern similar to the ring-elongated	Known system cancer

Only typical findings are reported.

AQP4+NMOSD, aquaporin-4-IgG-positive neuromyelitis optica spectrum disorders; DAVF, dural arteriovenous fistula; GFAP, glial-fibrillary acidic protein; MOGAD, myelin oligodendrocyte glycoprotein antibody-associated disease; MS, multiple sclerosis.

**Figure 3:**
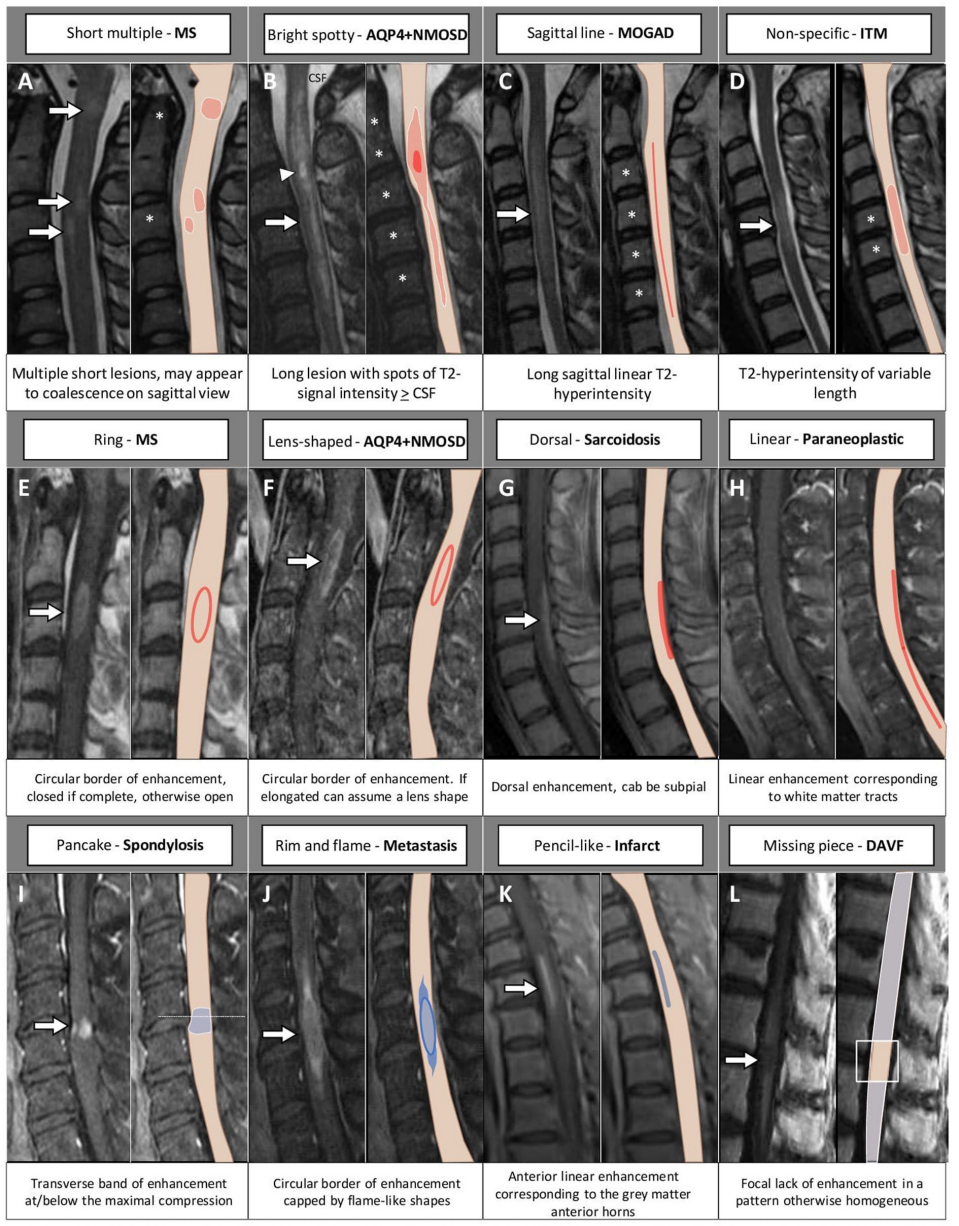
MRI and corresponding schematic representation of key findings on sagittal view of inflammatory myelopathies and their mimics. Each finding, labeled by a letter **(A–L)**, is shown on the most appropriate magnetic resonance imaging sequence (e.g., T2-weighted image in the top row; post-contrast T1-weighted image in the bottom rows), a brief description is provided. The extension of the T2-hyperintensities along consecutive vertebral segments is highlighted by asterisks; red color indicates inflammatory myelopathies, while blue color indicates their mimics. The arrowhead in **(B)** indicates a bright spotty lesion within a longitudinally extensive T2-hyperintensity. AQP4+NMOSD, aquaporin-4-IgG-positive neuromyelitis optica spectrum disorders; CSF, cerebrospinal fluid; DAVF, dural arteriovenous fistula; ITM, idiopathic transverse myelitis; MOGAD, myelin oligodendrocyte glycoprotein antibody-associated disease; MS, multiple sclerosis.

**Figure 4:**
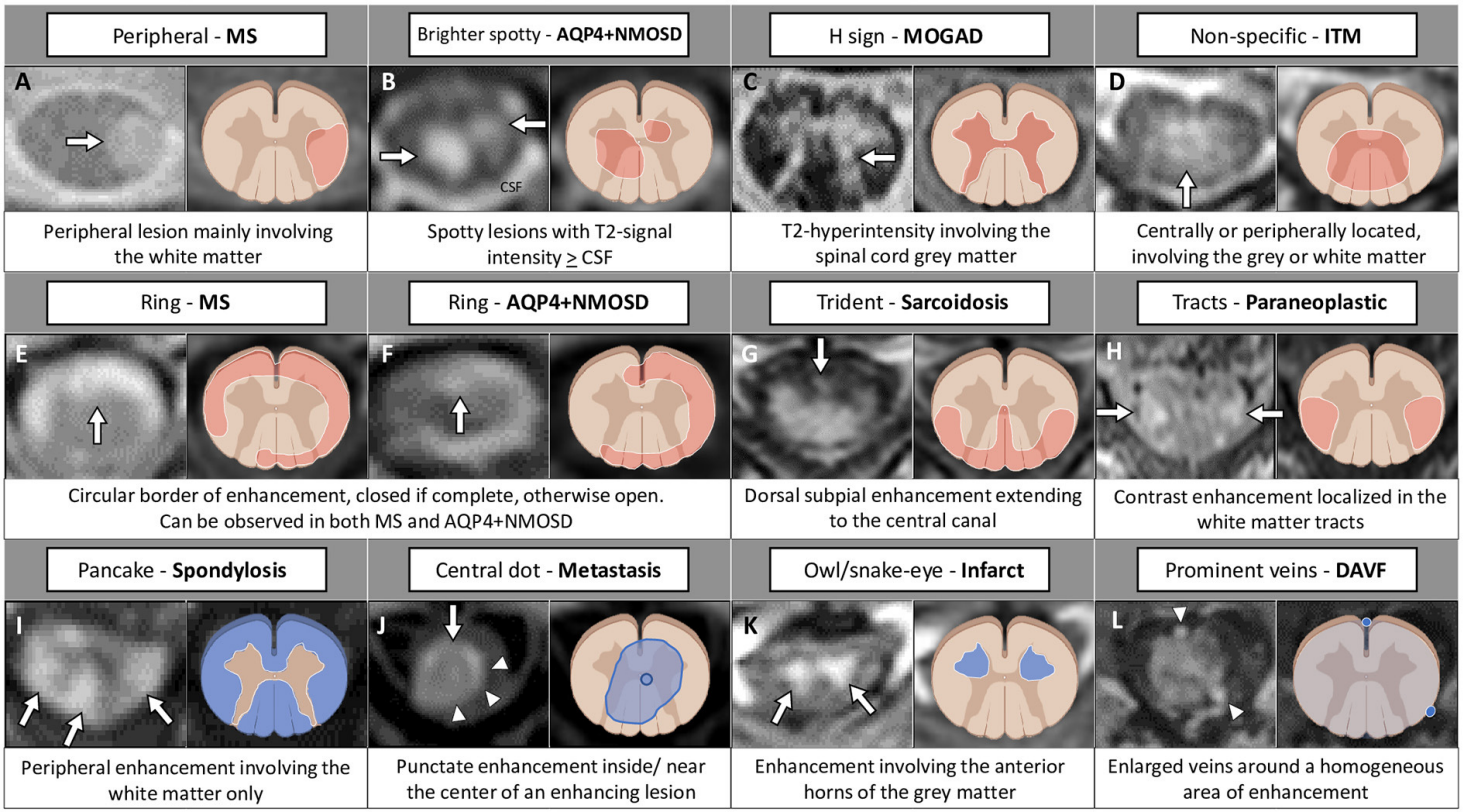
MRI and corresponding schematic representation of key findings on axial view of inflammatory myelopathies and their mimics. Each finding, labeled by a letter **(A–L)**, is shown on the most appropriate magnetic resonance imaging sequence (e.g., T2-weighted image in the top row; post-contrast T1-weighted image in the bottom rows), a brief description is provided. Red color indicates inflammatory myelopathies, while blue color indicates their mimics. The figure was created with BioRender.com. The arrowheads in **(J)** highlight a peripheral area of enhancement surrounding the central dot sign; in **(L)** indicate two veins visible around the area of enhancement. AQP4+NMOSD, aquaporin-4-IgG-positive neuromyelitis optica spectrum disorders; CSF, cerebrospinal fluid; DAVF, dural arteriovenous fistula; ITM, idiopathic transverse myelitis; MOGAD, myelin oligodendrocyte glycoprotein antibody-associated disease; MS, multiple sclerosis.

Figures 3, 4 provide a schematic representation of the key radiological signs associated with inflammatory myelopathies and their mimics on sagittal (Figure 3) and axial view (Figure 4).

***Spinal cord infarction***. Spinal cord infarction is one of the main mimics of inflammatory myelopathies, and the recognition of this entity is crucial for a prompt and appropriate treatment. The presence of a linear “pencil-like” strip of T2-hyperintensity with corresponding enhancement in the anterior horns of the spinal cord (“owl or snake-eye sign”) may indicate spinal cord infarct (98). However, this signal abnormality has also been reported in a similar proportion of patients with AQP4+NMOSD, therefore clinical findings must be accurately assessed (97). Evidence of concomitant infarct of one of the adjacent vertebral bodies strongly supports ischemia (114) but its frequency was low (4%) in some cohorts (115). Clinical presentation can provide useful clues: the onset is usually hyperacute (median time-to-nadir of 1 hour) (116) and lower motor neuron deficits such as flaccid paraparesis, areflexia, and mute plantar response prevail over upper motor signs in the early phase (“spinal shock”). In this circumstance, the presence of a clear trunk sensory level should raise the suspicion of central rather than peripheral injury. Back pain at the level of lesion location is another red-flag, (115) and an accurate medical history of the patient will reveal cardiovascular risk factors in two thirds of cases (114).

Recent findings support the measurement of neurofilament light chain in serum, together with the maximal area of the T2-hyperintensity in the cord, to discriminate among spinal cord ischemia, AQP4+NMOSD- or MOGAD-associated myelitis, and idiopathic transverse myelitis, since up to ten times higher values indicated vascular spinal cord injury (117).

***Nutritional deficits***. Subacute combined degeneration in the setting of vitamin B12 deficiency can mimic paraneoplastic longitudinally extensive myelopathies, because the T2-hyperintensity is usually symmetric and confined to the posterior or lateral white matter columns. Aside from laboratory abnormalities, including anemia, increased homocysteine, and low transcobalamin levels in serum, the lack of enhancement might be a useful red flag. Nitrous oxide exposure can also lead to a myelopathy with similar features, since it deactivates vitamin B12, and this more acute myelopathy can occasionally be accompanied by gadolinium enhancement, mimicking inflammatory or neoplastic etiologies (118). Copper deficiency has similar MRI characteristics, but preferentially involves the cervical segment extending to the central spinal cord (119).

***Spondylotic myelopathy***. In a patient with progressive clinical deficits, a fusiform T2-hyperintensity with a sagittal transverse band of enhancement with a width at least equal of height at or just below the level of the maximal spinal cord compression is observed in spondylotic myelopathy (“pancake sign”) (120). This may reflect focal breakdown of the blood spinal cord barrier (120). On axial view, it appears as a circumference of contrast enhancement involving the peripheral white matter and sparing the central gray matter. After surgical decompression, the enhancement reduces over time but can take months to years to resolve completely (120).

***Spinal dural arteriovenous fistula***. Spinal dural arteriovenous fistula is associated with a progressive myelopathy, with symptoms worsened by exercise, Valsalva, or postural changes and improved by rest. The typical demographic is older males but it can occur at any age and in younger patients congenital venous anomalies may be an explanation (121, 122). Since more than 90% of fistulae are located in the thoracolumbar spinal cord, T2-lesions are usually located in the thoracic cord with evidence of involvement of the conus in 80-95% of cases (114). A focal lack of enhancement (“missing-piece sign”) in an otherwise homogeneous pattern of contrast enhancement can be observed in spinal dural arteriovenous fistula possibly from differences in venous egress (98). Sagittal T2-weighted images can depict flow voids dorsal to the cord corresponding to the T2-hyperintensity, representing dilated perimedullary veins. However, sometimes these can be difficult to appreciate in the setting of a swollen spinal cord (2, 98). In any patient with a longitudinally extensive T2-lesion in the thoracic spine, spinal dural arteriovenous fistula should be considered in the differential diagnosis given it is treatable. The identification of this condition is challenging and up to half of patients are initially assigned an incorrect diagnosis of myelitis (123, 124), with potentially harmful effects, since steroid administration can worsen symptomatology (125).

***Spinal cord tumors***. Neoplastic conditions include both primary intramedullary spinal cord tumors and metastases. The most common primary neoplasm of the spinal cord in adults is ependymoma, whose MRI appearance is that of a long lesion with local spinal cord enlargement. The presence of a T1-hypointense cyst rostral or caudal to the T2-hyperintensity can be helpful in identifying ependymomas since it is absent in only 10% of cases. A T2-hypointense rim indicating hemosiderin deposit (“cap sign”) is observed in approximately 25% of patients (126). Enhancement is usually present, although with a homogeneous non-specific pattern (127). Astrocytomas are the most common primary neoplasm of the spinal cord in children, and may mimic MOGAD-associated spinal cord lesions, especially when located at the conus. Distinctive elements are represented by their excentric location (sometimes mimicking extra-axial neoplasms), the irregular margins indicating an infiltrative process (71), and prominent perilesional edema. Enhancement, when present, is heterogeneous. Sarcoidosis and AQP4+NMOSD represent the inflammatory myelopathies most often misdiagnosed as spinal cord malignancies (ependymomas in the case of AQP4+NMOSD) given their association with spinal cord swelling.

Intramedullary spinal cord metastases may mimic inflammatory lesions, since they present with a pattern of contrast enhancement similar to the elongated ring observed in AQP4+NMOSD. However, in the case of metastases, the center of the enhancing region is characterized by fainter contrast-enhancement than in the periphery. The top or the bottom of the rim is characterized by a “flame” appearance (“rim-and-flame sign”). The presence of the “rim” either the “flame” in isolation are 94% specific of paraneoplastic myelopathies, with an almost perfect specificity when observed in combination (128). The rim-and-flame sign can be accompanied by a punctate enhancement at or next to the center of the enhancing lesion, which identifies the “central dot sign”. This radiological aspect occurs in a minority of spinal cord metastasis (around 9%), in association with the complete rim-and-flame sign or with the rim sign alone (129). It represents a promising tool for the identification of patients with metastatic involvement of the spinal cord since it seems not to occur in patients with primary spinal cord tumors (129). Another aspect that may be considered is the mismatch between the length of the T2-hyperintensity and of gadolinium enhancement, since the former, likely due to spinal cord edema, is approximately 3.5 folds longer (130). When intramedullary metastasis is suspected, lung and breast imaging should be prioritized as these are the most common tumors that metastasize to the intramedullary spinal cord.

Among hematologic malignancies, intramedullary spinal cord lymphoma is a rare cause of myelopathy, often misdiagnosed as inflammatory despite the presence of a few atypical elements, including advanced age (around 60 years), constitutional symptoms, and back pain (60%) (131). Onset is almost invariably subacute or progressive, leading to wheelchair dependency in half of the patients in <1 year. Spinal cord imaging always reveals spinal cord T2-hyperintensities, which in most cases are multiple (64%) and involve the conus or cauda equina (57%). All lesions were reported with persistent gadolinium enhancement after 3 months from onset and had hypermetabolism at PET imaging, similar to sarcoidosis (131).

***Infectious diseases***. To conclude, it is worth to mention that infections may mimic inflammatory autoimmune myelopathies, including bacterial (e.g., tuberculosis, lyme, syphilis), viral (e.g., enteroviruses, West Nile virus, Varicella Zoster virus, human T lymphotropic virus type 1, human immunodeficiency virus [HIV], poliovirus), fungi and parasitic (e.g., schistosomiasis) etiologies. The discrimination between infectious and inflammatory myelopathies based on the sole imaging features is not possible, and other paraclinical exams, including CSF examination and laboratory tests may be more informative (132).

A recent history of travel in endemic regions, the presence of recent systemic symptoms such as fever, night sweats, and skin rashes, or individual risk factors (e.g., immunodeficiency or immunosuppression) might help to identify the underlying infection. Imaging may reveal longitudinally extensive lesions located in the spinal cord gray matter (enteroviruses, West Nile virus) (2) and posterior white matter tract involvement (HIV).

To note, 19% of MOGAD patients with flaccid myelitis were diagnosed with viral or postviral myelitis given the clinical and radiological overlap between MOGAD myelitis and the enterovirus D68 acute flaccid myelitis (34).

**Figure 5:**
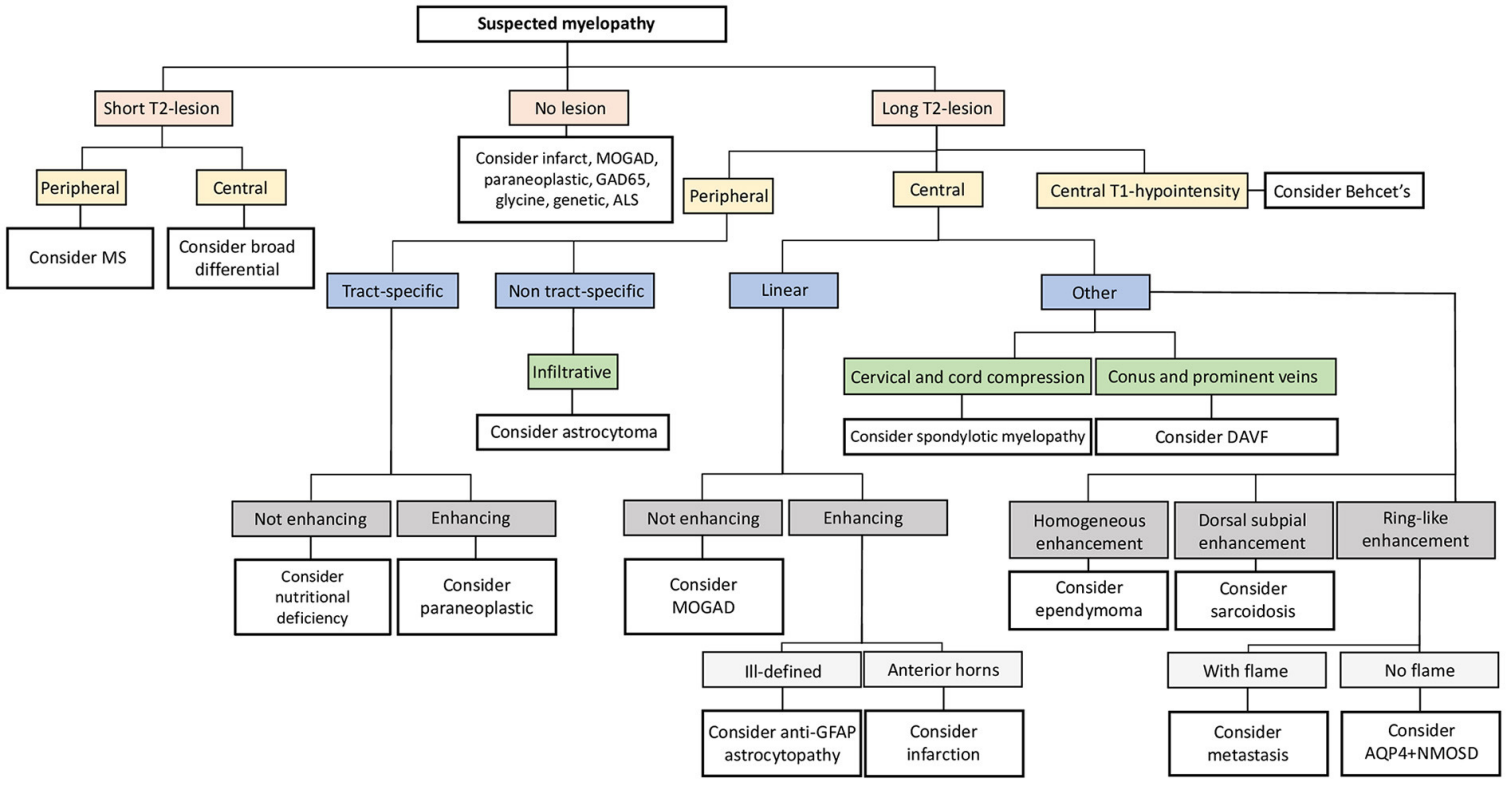
Neuroimaging approach to the differential diagnosis of suspected myelopathies. Flow-chart summarizing a possible diagnostic approach to suspected myelopathies from an imaging perspective. ALS, amyotrophic lateral sclerosis; AQP4+NMOSD, aquaporin-4-IgG-positive neuromyelitis optica spectrum disorders; DAVF, dural arteriovenous fistula; GAD65, glutamic acid decarboxylase-65; GFAP, glial-fibrillary acidic protein; MOGAD, myelin oligodendrocyte glycoprotein antibody-associated disease; MS, multiple sclerosis.

A flow-chart summarizing a proposal of a differential diagnostic approach to myelopathies (from an imaging perspective) is available as Figure 5. Please, note that, given the heterogeneity of infectious disorders, these are not considered in the algorithm, and must be thoroughly ruled out based on clinical presentation, patient's history, and laboratory findings.

## Other imaging techniques

Additional imaging techniques have been used to facilitate the differential diagnosis of myelopathies, although reports are still scarce and mainly limited to research setting.

### Magnetic resonance spectroscopy

1H-Magnetic resonance spectroscopy (1H-MRS) is an imaging technique able to measure the levels of highly concentrated metabolites such as the marker of neuronal mitochondrial metabolism N-acetyl-aspartate (NAA, used to indicate neuronal integrity), the acetylcholine precursor choline (Cho, indicating cellular membrane turnover), and the marker of astrocytes activation or proliferation myo-inositol (Myo). Peaks can be normalized by the voxel value of creatine and phosphocreatine (Cr, components of cellular membranes). Additional metabolites become visible in patients and encompass the main excitatory neurotransmitter glutamate/glutamine (Glx), and the lactate (marker of anaerobic metabolism).

So far, most studies using 1H-MRS to characterize inflammatory myelopathies focused on MS, where lesions showed increased levels of Myo, accompanied by high levels of Cho and Cr in the active phase, and reduced NAA in the chronic phases (133). Taken together these patterns suggested the presence of glial cells activation, with inflammatory infiltrates and possible myelin debris in active lesions, followed by chronic neuronal loss (133).

Interestingly, in contrast with MS, one case series suggested that Myo levels are decreased in spinal cord lesions of patients with AQP4+NMOSD compared to both MS and healthy controls, possibly reflecting the disease-specific astrocyte damage (134).

In the setting of the differential diagnosis of spinal cord lesions, glial tumors of the spinal cord such as the ependymoma have a metabolic profile similar to inflammatory MS lesions (e.g., reduction of NAA/Cr, increase of Cho/Cr, and increase of myo-inositol/Cr). However, in this case a lactate peak is also observed (135). Increased Cho/Cr levels were also observed in patients with spinal cord T2-hyperintensities concomitant with cervical spondylosis, but in this case without significant decrease of NAA/Cr (136).

### Positron emission tomography

18F-Fluorodeoxyglucose-positron emission tomography (FDG-PET) measures the metabolic activity by the local uptake of glucose. Evidence of hypermetabolism in the spinal cord may suggest a neoplastic rather than inflammatory cause, as it is more frequent and severe with neoplastic primary or secondary spinal cord lesions (81%) compared with inflammatory myelopathies (25%) (137). In line with this, a pilot study demonstrated decreased glucose-uptake of the spinal cord in patients with MS compared to healthy controls in the thoracic and lumbar segments (138).

An exception among inflammatory disorders is represented by sarcoidosis, where the rate (50%) and degree of spinal cord hypermetabolism similar to that of spinal cord tumors (137). FDG-PET imaging may be particularly useful in differentiating sarcoid- and AQP4+NMOSD-related longitudinally-extensive lesions, since glucose uptake is usually normal in AQP4+NMOSD, (46) although exceptions have been reported. However, the clinical use of this technique is limited by high cost and availability (139).

## Conclusions

MRI is a fundamental tool for the diagnostic assessment of inflammatory spinal cord lesions. Although the classical definition of longitudinally extensive and short myelitis can be useful for the identification of MS over different causes of autoimmune myelopathies, its utility is limited when discriminating between other immune-mediated or non-immune mediated myelopathies.

In this scenario, novel MRI signs and enhancement patterns are an excellent tool for clinicians to guide differential diagnosis and select appropriate diagnostic tests. Recognition of these radiological aspects will help clinicians achieve prompt diagnosis and earlier disease-specific treatment, which will ultimately improve patient outcomes.

Other imaging techniques, able to underpin the metabolic profile and activity of spinal cord lesions are promising as well, although additional studies on larger cohorts are warranted.

## Author contributions

LC and EF review concept, design, and drafted the manuscript and figures. ES, MR, MF, and SP revised the manuscript for intellectual content. All authors contributed to the article and approved the submitted version.

## Funding

EF has received funding from the NIH (R01NS113828).

## Conflict of interest

The authors declare that the research was conducted in the absence of any commercial or financial relationships that could be construed as a potential conflict of interest.

## Publisher's note

All claims expressed in this article are solely those of the authors and do not necessarily represent those of their affiliated organizations, or those of the publisher, the editors and the reviewers. Any product that may be evaluated in this article, or claim that may be made by its manufacturer, is not guaranteed or endorsed by the publisher.
